# Disseminated Histoplasmosis with Miliary Histoplasmosis, Neurohistoplasmosis, and *Histoplasma capsulatum* Bacteremia in Probable Neurosarcoidosis

**DOI:** 10.1155/2018/3162403

**Published:** 2018-12-11

**Authors:** Peter V. Bui

**Affiliations:** Department of Emergency Medicine, University of Michigan, Ann Arbor, MI, USA

## Abstract

**Introduction:**

Neurosarcoidosis, either isolated or as part of systemic sarcoidosis, is an uncommon entity and has diagnostic uncertainty. Treatment for neurosarcoidosis can increase the risk of infections, including fungal infections such as disseminated histoplasmosis. Neurosarcoidosis may further predispose patients to infections of the central nervous system.

**Case Presentation:**

A 54-year-old male with a history of probable neurosarcoidosis on methotrexate and infliximab presented with encephalopathy, hypoxia, and reported fevers. The patient was found to have disseminated histoplasmosis involving the lungs (miliary histoplasmosis), central nervous system (neurohistoplasmosis), and bloodstream. The *Histoplasma capsulatum* infection was treated with amphotericin and then voriconazole.

**Discussion:**

Patients with neurosarcoidosis are suspected to have blood-brain barrier dysfunction. Lumbar puncture should be considered as part of initial investigative studies for infection. Empiric antimicrobial therapy for a patient with neurosarcoidosis on immunosuppressive agents may need to include antifungal agents.

## 1. Introduction

Neurological symptoms occur in approximately 5% of patients with sarcoidosis [1–3]. Because of the complexity involved in diagnosing neurosarcoidosis, particularly isolated neurosarcoidosis, Zajicek et al. proposed criteria for definite, probable, and possible neurosarcoidosis (Table 1) [4].

For patients with sarcoidosis, risk factors for severe infections, defined as infections leading to hospitalization or death or requiring intravenous antibiotics, included neurologic symptoms, cardiac symptoms, or immunosuppressant use [5]. These infections can include fungal infections, even disseminated histoplasmosis [5–9]. We present the case of a patient diagnosed with probable neurosarcoidosis who subsequently developed disseminated histoplasmosis with miliary histoplasmosis, central nervous system histoplasmosis (neurohistoplasmosis), and *Histoplasma capsulatum* bacteremia. In our review of the English medical literature, we did not identify any additional cases of extensive multisystem infection with *Histoplasma capsulatum* in a patient with neurosarcoidosis.

## 2. Case Presentation

A 54-year-old male with a history of probable neurosarcoidosis, lymphocytic leptomeningitis, seizures, cirrhosis, and possible ethanol use disorder presented with encephalopathy, hypoxia, and several weeks of reported intermittent fevers. For the treatment of neurosarcoidosis, he was taking mycophenolate 1000 mg orally twice a day and last received an infliximab infusion the month prior to presentation.

Approximately seventeen months prior to presentation, the patient underwent extensive investigation for ataxia, near syncope, and encephalopathy, during which he received empiric treatment for encephalitis with corticosteroids, although herpes simplex virus studies were negative. Magnetic resonance imaging of the brain and spine from the cervical spinal cord to the lumbar spinal cord found leptomeningeal enhancement. Cerebral spinal fluid (CSF) found 24 white blood cells/mm^3^ (reference range 0–5 cells/mm^3^) with 83% lymphocytes (reference range 0–60%), 8 red blood cells/mm^3^ (reference range 0–0 cells/mm^3^), glucose of 35 mg/dL (reference range 40–70 mg/dL), protein of 247.6 mg/dL (reference range 15–45 mg/dL), and no oligoclonal bands. Biopsies of the brain and meninges found leptomeningeal fibrosis with prominent arachnoid cap cells. Chest imaging and fluorodeoxyglucose-positron emission tomography found nonspecific pulmonary nodules and hilar lymphadenopathy. Additional unremarkable studies included bone marrow biopsies, lung biopsies, and sinus biopsies. No infectious pathogens, including *Histoplasma*, or neoplastic processes were identified.

Eleven months prior to presentation, the patient presented with encephalopathy. He was thought to have lymphocytic leptomeningitis. Serum and CSF angiotensin-converting enzymes (ACE) were normal. CSF studies found 21 white blood cells/mm^3^ (no reference range) with 91% lymphocytes (no reference range), 1 red blood cell/mm^3^ (no reference range), glucose of 39 mg/dL (50–70 mg/dL), and protein of 188 mg/dL (15–45 mg/dL). Investigations for infectious pathogens, including serum and CSF fungal studies such as for histoplasmosis, and neoplastic processes were negative. He received intravenous immunoglobulins and corticosteroids for an unclear autoimmune disease. A month later, his neurology providers thought his symptoms and studies were consistent with probable neurosarcoidosis and proceeded with treatment using mycophenolate and infliximab. Six months prior to presentation, he developed keratitis and uveitis.

**Figure 1:**
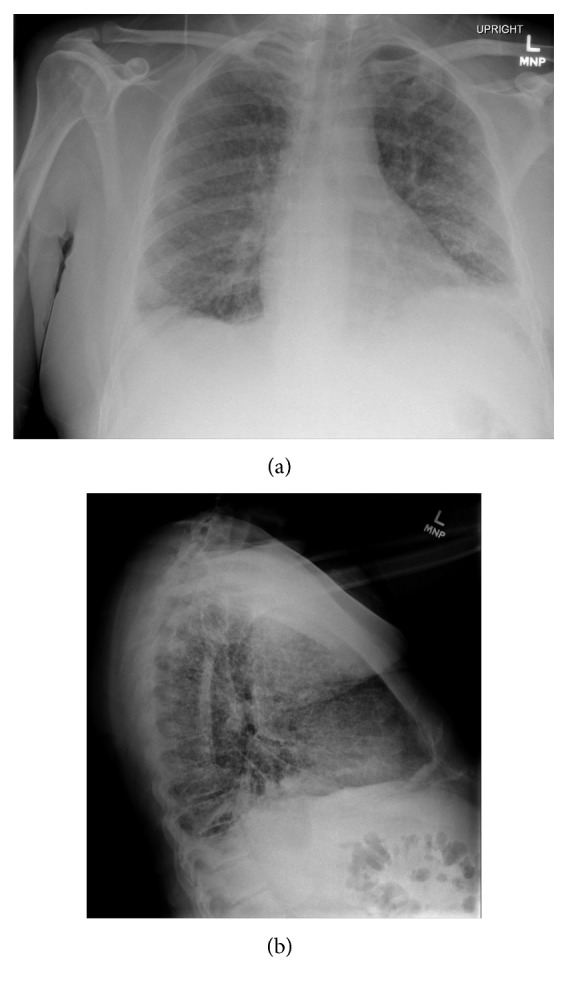
Conventional chest radiography obtained at the initial presentation found a diffuse miliary pattern. (a) Posterior-anterior view. (b) Lateral view.

Vital signs at presentation were temperature of 36.4°C, blood pressure of 80 mmHg/52 mmHg, heart rate of 93 beats per minute, respiratory rate of 24 breaths per minute, and oxygen saturation of 91% on 10 liters per minute of supplemental oxygen. Conventional chest radiography (Figure 1) found a diffuse miliary pattern, which was also identified on subsequent computed tomography of the chest. CSF studies found 1 white blood cell/mm^3^ (no reference range) with 95% lymphocytes (no reference range), 1 red blood cell/mm^3^ (no reference range), glucose of 28 mg/dL (50–70 mg/dL), and protein of 53 mg/dL (15–45 mg/dL).

The patient received empiric vancomycin, piperacillin/tazobactam, and amphotericin for possible bacterial and fungal infections. He underwent endotracheal intubation for acute hypoxic respiratory failure and was extubated approximately one week later. Urine, serum, and CSF antigen studies were positive for histoplasmosis and blastomycosis, but the positive studies for blastomycosis were thought to be secondary to cross-reactivity. Respiratory culture obtained via bronchoscopy grew *Histoplasma capsulatum*. Fungal blood culture grew *Histoplasma capsulatum*. Bone marrow biopsy, obtained after the administration of antibacterial and antifungal agents, found hemophagocytosis that was thought to be consistent with the diagnosis of disseminated histoplasmosis, although the bone marrow culture had no growth of pathogens. For treatment of disseminated histoplasmosis, he completed a course of amphotericin, was briefly on itraconazole, and was transitioned to voriconazole, on which he was eventually discharged after a month-long hospitalization. *Pneumocystis jirovecii* polymerase chain reaction study of the bronchoalveolar lavage was positive for which he received a complete course of trimethoprim-sulfamethoxazole, although suspicion for pneumocystis pneumonia was low. The patient's hospital course was complicated by acute kidney injury, aspiration pneumonia, and *Pseudomonas aeruginosa* pneumonia.

## 3. Discussion

This patient was diagnosed with probable neurosarcoidosis. Evidence of systemic sarcoidosis in this patient included hilar lymphadenopathy and a history of uveitis and keratitis, although these findings do not conform to all the proposed criteria of Zajicek et al. for the diagnosis of neurosarcoidosis [4, 10]. Central nervous system studies suggestive of neurosarcoidosis included leptomeningeal enhancement, elevated proteins in the CSF, and CSF lymphocytic pleocytosis [2, 4, 11]. No oligoclonal bands were found. Serum ACE, which can be elevated in sarcoidosis, was normal. [4] CSF ACE was normal but lacks clinical utility in the diagnosis of neurosarcoidosis [12, 13]. Extensive studies during multiple hospitalizations did not find alternative infectious and neoplastic pathologies to fully account for the patient's symptoms and findings, thereby lending credence to neurosarcoidosis as a diagnosis of exclusion. Additionally, the patient had clinical improvement on methotrexate and infliximab and previously corticosteroids, which are typical treatments for neurosarcoidosis [2, 11, 14].

Our patient developed disseminated histoplasmosis involving the central nervous system, lungs, and bloodstream. Taking methotrexate and infliximab had predisposed him to disseminated histoplasmosis. Furthermore, the blood-brain barrier is thought to be dysfunctional in neurosarcoidosis, a factor which likely contributed to neurohistoplasmosis in our patient [4]. Clinicians should consider fungal studies and empiric antifungal treatment in patients with neurosarcoidosis on immunosuppressants. In a patient with neurosarcoidosis with suspected infection, because of the blood-brain barrier dysfunction, early lumbar puncture may be beneficial to confirm or exclude central nervous system involvement even if another infectious source is identified.

## Figures and Tables

**Table 1:** Proposed diagnostic criteria for neurosarcoidosis [4].

Definite	Clinical presentation suggestive of neurosarcoidosis with exclusion of other possible diagnoses and the presence of positive nervous system histology

Probable	Clinical syndrome suggestive of neurosarcoidosis with laboratory support for central nervous system inflammation (elevated levels of cerebrospinal fluid protein and/or cells, the presence of oligoclonal bands, and/or magnetic resonance imaging evidence compatible with neurosarcoidosis) and exclusion of alternative diagnoses together with evidence for systemic sarcoidosis (either through positive histology, including Kveim test and/or at least two indirect indicators from gallium scan, chest imaging, and serum angiotensin converting enzyme)

Possible	Clinical presentation suggestive of neurosarcoidosis with exclusion of alternative diagnoses where the above criteria are not met

## References

[1] Baughman R. P., Teirstein A. S., Judson M. A. (2001). Clinical characteristics of patients in a case control study of sarcoidosis. *American Journal of Respiratory and Critical Care Medicine*.

[2] Fritz D., van de Beek D., Brouwer M. C. (2016). Clinical features, treatment and outcome in neurosarcoidosis: systematic review and meta-analysis. *BMC Neurology*.

[3] Stern B. J., Krumholz A., Johns C., Scott P., Nissim J. (1985). Sarcoidosis and its neurological manifestations. *Archives of Neurology*.

[4] Zajicek J. P., Scolding N. J., Foster O. (1999). Central nervous system sarcoidosisdiagnosis and management. *QJM*.

[5] Duréault A., Chapelon C., Biard L. (2017). Severe infections in sarcoidosis. *Medicine*.

[6] Badesha P. S., Saklayen M. G., Hillman N. (1997). Diffuse *Histoplasmosis* in a patient with sarcoidosis. *Postgraduate Medical Journal*.

[7] Baughman R. P., Lower E. E. (2005). Fungal infections as a complication of therapy for sarcoidosis. *QJM: An International Journal of Medicine*.

[8] Israel H. L., DeLamater E., Sones M., Willis W. D., Mirmelstein A. (1951). An investigation of the relationship of chronic disseminated histoplasmosis and sarcoidosis. *Bulletin of the New York Academy of Medicine*.

[9] Tebib J. G., Piens M. A., Guillaux M., Colomb D., Garin J. P., Tete R. (1988). Sarcoidosis possibly predisposing to disseminated histoplasmosis. *Thorax*.

[10] Koczman J. J., Rouleau J., Gaunt M., Kardon R. H., Wall M., Lee A. G. (2009). Neuro-ophthalmic sarcoidosis: the University of Iowa experience. *Seminars in Ophthalmology*.

[11] Cação G., Branco A., Meireles M. (2017). Neurosarcoidosis according to Zajicek and Scolding criteria: 15 probable and definite cases, their treatment and outcomes. *Journal of the Neurological Sciences*.

[12] Bridel C., Courvoisier D. S., Vuilleumier N., Lalive P. H. (2015). Cerebrospinal fluid angiotensin-converting enzyme for diagnosis of neurosarcoidosis. *Journal of Neuroimmunology*.

[13] Khoury J., Wellik K. E., Demaerschalk B. M., Wingerchuk D. M. (2009). Cerebrospinal fluid angiotensin-converting enzyme for diagnosis of central nervous system sarcoidosis. *Neurologist*.

[14] Wegener S., Linnebank M., Martin R., Valavanis A., Weller M. (2014). Clinically isolated neurosarcoidosis: a recommended diagnostic path. *European Neurology*.

